# The Interplay between Meningeal Lymphatic Vessels and Neuroinflammation in Neurodegenerative Diseases

**DOI:** 10.2174/1570159X21666221115150253

**Published:** 2022-12-15

**Authors:** Junmei Chen, Yaru Pan, Qihua Liu, Guangyao Li, Gongcan Chen, Weirong Li, Wei Zhao, Qi Wang

**Affiliations:** 1 Science and Technology Innovation Center, Guangzhou University of Chinese Medicine, Guangzhou, 510000, China;; 2 Institute of Clinical Pharmacology, Guangzhou University of Chinese Medicine, Guangzhou, 510000, China;; 3 Clinical Medical College of Acupuncture Moxibustion and Rehabilitation, Guangzhou University of Chinese Medicine, Guangzhou, 510000, China

**Keywords:** Meningeal lymphatic vessels, neuroinflammation, interplay, neurodegenerative diseases, neurodegeneration, interaction

## Abstract

Meningeal lymphatic vessels (MLVs) are essential for the drainage of cerebrospinal fluid, macromolecules, and immune cells in the central nervous system. They play critical roles in modulating neuroinflammation in neurodegenerative diseases. Dysfunctional MLVs have been demonstrated to increase neuroinflammation by horizontally blocking the drainage of neurotoxic proteins to the peripheral lymph nodes. Conversely, MLVs protect against neuroinflammation by preventing immune cells from becoming fully encephalitogenic. Furthermore, evidence suggests that neuroinflammation affects the structure and function of MLVs, causing vascular anomalies and angiogenesis. Although this field is still in its infancy, the strong link between MLVs and neuroinflammation has emerged as a potential target for slowing the progression of neurodegenerative diseases. This review provides a brief history of the discovery of MLVs, introduces *in vivo* and *in vitro* MLV models, highlights the molecular mechanisms through which MLVs contribute to and protect against neuroinflammation, and discusses the potential impact of neuroinflammation on MLVs, focusing on recent progress in neurodegenerative diseases.

## 1 INTRODUCTION

Meningeal lymphatic vessels (MLVs) drain the cerebrospinal fluid (CSF) and interstitial fluid (ISF) into the peripheral lymph nodes [1-6]. They help maintain fluid dynamics and regulate the levels of toxic proteins in brain fluids, thereby influencing cognitive function [3]. Since the resurgence of interest in MLVs in 2015 (Table **1**), the critical role of MLVs in neurodegenerative diseases (NDs) has become a major research topic [1, 4, 5, 7]. Compelling evidence suggests that MLVs contribute to cognitive decline in Alzheimer’s disease (AD) [3, 8, 9], Parkinson’s disease (PD) [10], multiple sclerosis (MS) [11], and stroke [12-14], and the use of MLV-modifying therapy to change the course of disease development in patients with NDs has a very promising future [4, 15, 16].

Neuroinflammation is a key pathologic feature of NDs [17]. In AD, PD, and amyotrophic lateral sclerosis, neuroinflammation is characterized by neurotoxic self-aggregation proteins, which trigger neuroinflammatory cascade reactions that are regulated by microglia and astrocytes [18]. The infiltration of specific lymphocytes and macrophages into the brain parenchyma, accompanied by severe glial reaction, influences the development of MS and stroke [18, 19]. Therefore, in most NDs, inflammatory mediators released by activated glia perpetuate the inflammatory cycle by activating additional microglia and astrocytes, stimulating their proliferation and causing the release of extended inflammatory factors, which accelerates the progression of NDs [20]. Epidemiological studies and clinical trials have also revealed that inflammation-related gene expression varies as NDs progress [21-24].

MLV dysfunction in NDs can induce numerous stimuli that trigger neuroinflammation, including neurotoxic proteins, extravasated erythrocytes, and glial activation [3, 25, 26]; this strongly indicates that dysfunctional MLVs are involved in neuroinflammation [27, 28]. It is tempting to assume that MLVs aggravate neuroinflammation, but this assumption could be overly simplistic. According to emerging evidence, MLVs can have detrimental effects in some NDs and beneficial effects in others. Furthermore, neuroinflammation can impact MLVs by inducing angiogenesis and cerebrovascular anomalies [3, 8-11, 14, 26, 29-32]. Therefore, the interplay between MLVs and neuroinflammation has emerged as an important and interesting topic. This interplay is a promising novel target for improving cognitive ability and clinical outcomes in NDs [33-36].

This review begins by introducing the anatomy and function of MLVs and providing an analysis of *in vitro* and *in vivo* models of MLVs employed to study NDs. It emphasizes the pro- and anti-inflammatory effects of MLVs and the influence of neuroinflammation on MLV structure and function. This review presents a detailed discussion on the relationship between MLVs and neuroinflammation to fully recapitulate the potential mechanism of the interaction between MLVs and neuroinflammation in NDs.

## 2 ANATOMY AND FUNCTION OF MLVs

### 2.1 Anatomic Location

**Fig. (1):**
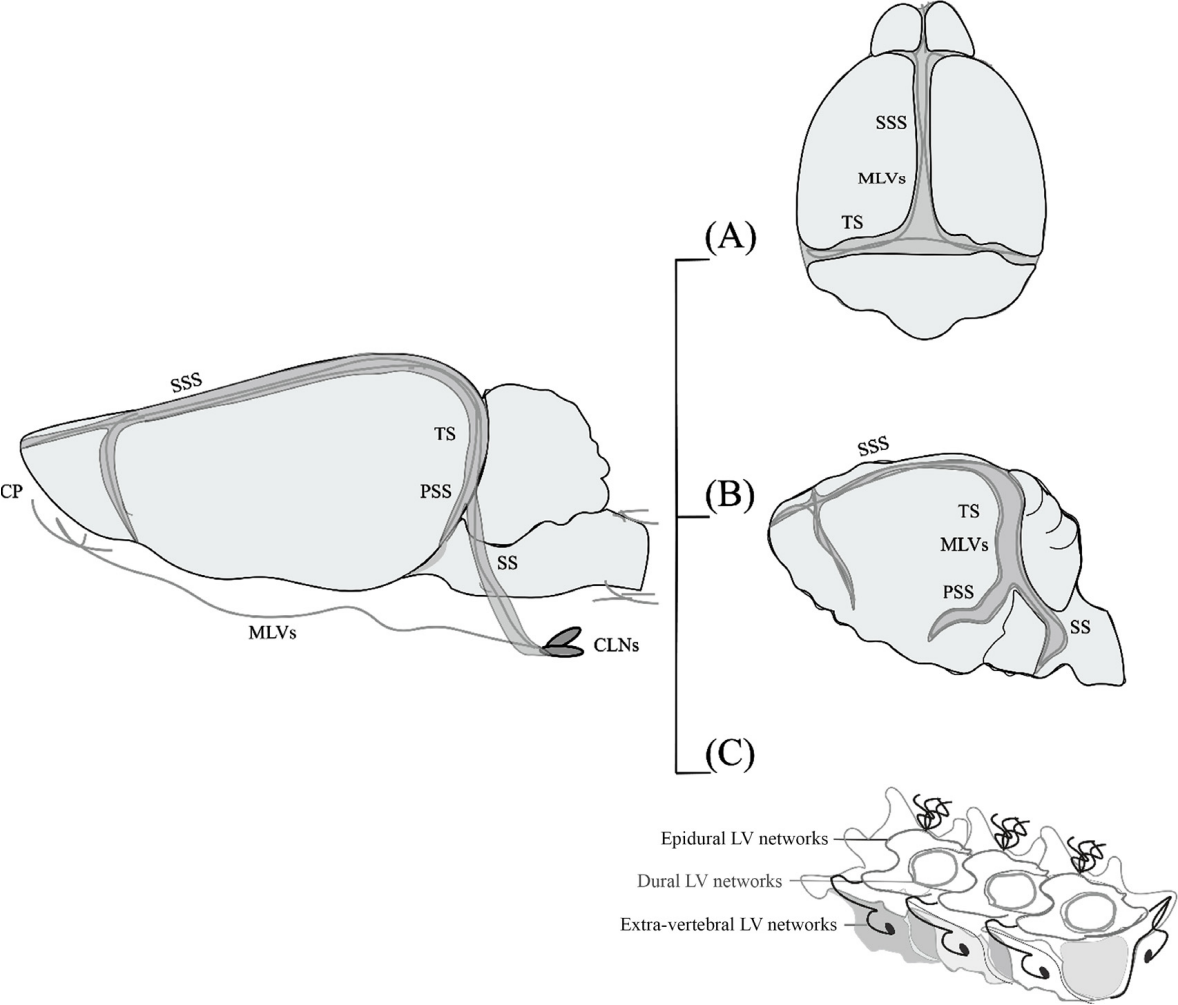
Scheme of MLV network in rodents. (**A**) Dorsal MLVs. (**B**) Basal MLVs. (**C**) Vertebral MLVs. CLNs: cervical lymph nodes; CP: cribriform plate; LV: lymphatic vessels; MLVs: meningeal lymphatic vessels; PSS: petrosquamosal sinus; SS: sigmoid sinus; SSS: superior sagittal sinus; TS: transverse sinus.

The meninges are composed of three distinct layers. The inner layer, known as the pia mater, is located near the nerve tissue of the brain and spinal cord. The subarachnoid gap separates the vascularized pia mater from the second avascular layer, the arachnoid mater. The arachnoid mater is firmly attached to the dura mater, the third meningeal layer. The dura is directly attached to the bone structures surrounding the brain; in the spinal cord, it is attached to the bone through the epidural space. This layer is vascularized, and unlike the other layers, it is endowed with MLVs. Recent research has visualized the distinct anatomical location of MLVs in different regions of the central nervous system (CNS) in rodents to characterize their morphological features (Fig. **1**). Dorsal MLVs appear to originate in both eyes, track above the olfactory bulb, run parallel to the superior sagittal sinus and transverse sinus, and connect to superficial and deep cervical lymph nodes (dCLNs) [1]. Basal MLVs travel along the petrosquamosal and sigmoid sinuses and communicate with the extracranial lymphatic vessels *via* the skull foramina [6]. Vertebral MLVs, which are primarily found above the dura mater in the epidural space, form metameric vertebral circuits that connect to lymph nodes, the thoracic duct, peripheral sensory nerves, and sympathetic ganglia [58]. MLVs have also been found in the cribriform plate and nasal mucosa, where they attach to superficial cervical lymph nodes [11, 55]. Interestingly, some evidence suggests that MLVs exist in the pituitary and communicate with perinasal tissue [59]. However, this requires further validation, as the prevailing opinion is that the brain parenchyma does not contain lymphatic vessels. Moreover, structural and functional non-aluminized lymphatic endothelial cells in the mammalian leptomeninges suggest that MLVs are present in the mammalian leptomeninges in addition to the dura mater [60].

The advent of novel, clinically available, and noninvasive magnetic resonance imaging methods to visualize MLVs has enabled clinical studies to shed light on the physiological and pathological aspects of MLVs in humans [46, 47, 49, 54]. The parasagittal dura has been identified as a link between human brain tissue and dural MLVs, revealing the pathway of molecular transport from the CSF to the dural MLVs [49]. Direct connections between the meningeal lymphatic fluid and dCLNs *via* multiple neural foramina and along the skull base structures have been visualized in humans [61]. In addition, patients with idiopathic PD exhibited significantly reduced lymphatic flow through the MLVs along the superior sagittal sinus and sigmoid sinus compared with patients with atypical Parkinsonian disorders, as well as a notable delay in dCLN perfusion [54].

### 2.2 Structural Characteristics and Functions

#### 2.2.1 CSF and Macromolecule Drainage

The CSF is constantly produced by the choroid plexus and flows through the CNS *via* the internal ventricles, subarachnoid space, cisterns, and perivascular spaces of cerebral arteries and veins [14]. Rodents have several CSF outflow pathways, namely the olfactory nerve through the cribriform plate; the trigeminal, glossopharyngeal, vagal, and spinal accessory nerves; and MLVs [62]. MLVs function physiologically as a drainage route for CSF and macromolecules to the peripheral lymph nodes [1, 3, 6]. The ability of the MLVs to drain CSF and macromolecules varies from region to region, and this variation is related to the structural characteristics of the MLVs. Basal MLVs are more likely than dorsal MLVs to be the most critical contributor to CSF drainage because the capillaries in basal MLVs have a loose arachnoid barrier and are located close to the subarachnoid space within a much thinner dura layer [6]. Furthermore, basal MLVs exhibit features of capillaries and collecting lymphatic vessels, including zipper-like junctions, lymphatic valves, and a lack of smooth muscle cell coverage [6]. These features allow them to perform both CSF uptake and transport. A major risk factor of MLV dysfunction is aging, which damages brain perfusion and reduces neurotoxic clearance [3, 63]. Increased meningeal lymphatic drainage in aged mice facilitates the clearance of CSF/ISF macromolecules from the brain, thereby enhancing cognitive performance [3]. The association between aging and MLVs may be responsible for some aspects of neuroinflammation-associated NDs.

However, the degree to which MLV drainage contributes to CSF outflow as a whole is still debatable [64]. Recent research has indicated that MLV drainage is vital for CSF elimination [3, 65]; however, using dynamic *in vivo* and *ex vivo* imaging of near-infrared fluorescently-labeled albumin of whole tissues, Brady M and colleagues detected less CSF elimination *via* the dural sinuses than *via* the nasal route across the cribriform plate and spinal subarachnoid space, implying that MLVs may not be the primary CSF outflow pathway in rodents [66]. These contradictory results could be attributable to the use of different imaging techniques. The related research topics in this area are interesting but challenging; therefore, they require further investigation.

#### 2.2.2 Immune Cell Drainage

The dura layer of the meninges contains a large population of immune cells [67, 68]. Sessile macrophages produce major histocompatibility complex molecules, implying that they can communicate with T cells and present antigens [68, 69]. IgA-producing plasma cells and T lymphocytes cluster in the venous sinuses and are thought to be adaptive components of CNS immune surveillance [70, 71]. MLVs act as key immune cell drainage pathways and regulate meningeal pro- and anti-inflammatory homeostasis. The MLV-mediated immune cell drainage is dependent on CC chemokine receptor 7 (CCR7) [11, 72, 73], which is essential for leukocyte-homing and the regulation of adaptive immunity and tolerance [74-76]. Different regions of MLVs may drain immune cells independently; therefore, different regions may play different roles in disease development, especially in the progression of chronic neuroinflammation [11]. Notably, vertebral MLVs may act as a critical immune surveillance interface between the CNS and peripheral organs [58]. Under physiological conditions, perilymphatic dendritic immune cells migrate to the cervical, mediastinal, and renal/lumbar lymph nodes from the cervical, thoracic, and lumbar areas, enhancing lymphocyte activation against specific pathogens or antigens. Vertebral MLVs may serve as prospective targets for NDs by accelerating the spread of peripheral infections across the spinal canal to the brain tissues.

Whereas MLVs likely play an active immunological role, recent evidence demonstrates that the dura plays a relatively passive role in both acute and chronic autoimmune diseases compared with the leptomeninges. The possible mechanisms are as follows [77]: first, effector T cells adhere more weakly to the dural endothelium than the leptomeningeal layers, and second, antigen-presenting cells present myelin and neuronal autoantigens less efficiently, and the activation of autoreactive T cells is lower in the dural layers than in the leptomeningeal layers. Therefore, the manipulation of MLVs to regulate neuroinflammation should be approached with caution [77].

#### 2.2.3 Differences Between Mice and Humans

Human MLVs and mouse MLVs differ in several ways. Unlike mouse MLVs, which are often present in pairs along the dural sinus and have a diameter of 20-30 μm [1, 5], human MLVs are typically present in clusters of more than five vessels with a diameter range varying widely from 19 to 470 μm [78]. MLV drainage diminishes with age in both mice and humans [3, 6, 61]; however, MLVs have been observed to vary with gender in humans [79, 80] but not in mice [1]. Despite the differences between MLVs in humans and mice, research on MLVs using mouse models may still contribute greatly to the field. According to a recent study, dural lymphatic CSF drainage circuits discovered in mice resemble the perisinusal lymphatic drainage circuits mapped in the human dura mater by magnetic resonance imaging [80]. Therefore, mice may be an excellent model for predicting the pathophysiological role of MLVs and testing lymphatic-targeted drugs in neurological diseases [80].

## 3 ASSESSING MLV FUNCTION

### 3.1 *In vivo* Models

#### 3.1.1 Pharmacological Models

Photodynamic therapy (PDT) is the most common pharmacological method used in MLV *in vivo* models. This therapeutic modality uses photosensitizers excited by a specific wavelength of light to produce molecular oxygen and exert a cell-killing effect [81]. To cause a specific type of lymphatic cell damage—lymphatic cell ablation—the photodynamic drug verteporfin is used in conjunction with a laser system that delivers a stable power output at a wavelength of 689 ± 3 nm [82]. During this process, interactions between verteporfin and light generate highly reactive singlet oxygen, which reacts with the surrounding solution components and initiates apoptosis and necrosis, destroys the vasculature, and stimulates the immune response to induce damage to and death of the target tissue [83]. PDT-based lymphatic cell ablation has been identified as an effective method for damaging MLV drainage of CSF, macromolecules, and immune cells in mice. For instance, dorsal MLV ablation impaired CSF influx, perfusion, and molecular diffusion, as well as ISF efflux, in the brain parenchyma, worsening the impairment of spatial learning and memory [3]. However, it is not known whether the PDT method is equally efficient for MLV ablation in other brain regions. Specific validation for MLV ablation in different brain regions should be thoroughly investigated to avoid off-target effects [3, 84]. Furthermore, most studies have employed mice as PDT models [3, 85]. This is most likely because of the operating procedure and observation process. Zebrafish are also a promising new model for studying the development of MLVs given their optical clarity and experimental advantages [52]. Recently, a study on PDT-based ischemic stroke utilized zebrafish as a disease model [13]; the success of this model indicates that future studies could establish a PDT-based MLV ablation model using zebrafish.

Because MLVs in adult mice are uniquely dependent on continuous vascular endothelial growth factor C (VEGF-C)/vascular endothelial growth factor receptor (VEGFR) 3 signaling, the VEGFR tyrosine kinase inhibitor [2, 14, 25, 86] and the VEGF-C trap [2] have also been used to establish an MLV *in vivo* model. Sunitinib is a VEGFR tyrosine kinase inhibitor that is used in cancer patients to target tumor angiogenesis. A two-week 60 mg/kg sunitinib treatment resulted in significant regression of parasinusoidal lymphatic vessels in mice, particularly in the superior sagittal sinus region [2]. Intracisternal injection of adeno-associated viral vectors encoding soluble VEGFR3, which encodes the mouse VEGF-C/D trap, resulted in the complete absence of MLVs around the dural sinuses, with some lymphatic vessels remaining in the basal part of the skull and around the spinal cord [2]. The mLV characteristics of these models should be considered when assessing the effects of meningeal lymphatic loss of function.

#### 3.1.2 Transgenic Mouse Models

Transgenic mice with lymphatic vessel deficiency are ideal research tools for studying MLV dysfunction [3, 5]. These mouse models exhibit specific pathological features of lymphatic vessel dysfunction, and studies have used them as *in vivo* models to demonstrate the relationship between MLVs and macromolecule drainage, typically by measuring the clearance of macromolecules from the brain and their drainage to the periphery. For example, in K14-VEGFR-3-Ig transgenic mice, MLVs are absent in both the superior and basal parts of the skull [5], and labeled tau is retained in the brain parenchyma with delayed clearance to the periphery [9]. The MLV deficiency in K14-VEGFR-3-Ig mice is attributed to its impaired VEGF-C/VEGFR3 signaling pathway. Specifically, this impairment involves the overexpression of a chimeric protein consisting of the ligand-binding portion of the extracellular part of VEGFR-3, which binds to the Fc domain of the immunoglobulin γ-chain to neutralize the activity of VEGF-C under the keratin-14 promoter [87]. However, in adult K14-VEGFR-3-Ig mice, lymphatic vessels may regrow in an abnormal pattern [87]. When using this model, the age of the mouse should be considered.

#### 3.1.3 Surgical Models

Meningeal lymphatic drainage can be disrupted by physically blocking the anatomical connection between the MLVs and the cervical lymph nodes. Surgical ligation of vessels afferent to the dCLNs [3], resection of dCLNs [1], and removal of superficial cervical lymph nodes [14] are the three most common surgical models for MLVs. These approaches effectively block CSF drainage and impair cognitive function in various disease models, including AD [3, 8], PD [10], and cerebral ischemic stroke [44].

#### 3.1.4 Model Comparison

Current evidence suggests that pharmacological, surgical, and transgenic MLV models have similar impacts on CSF drainage (Table **2**). However, these models have different characteristics, including the mechanisms of MLV dysfunction, which may affect the assessment of MLV function. First, the vascular structure of MLVs is damaged in pharmacological and transgenic models but may remain intact in surgical models. Thus, pharmacological and transgenic models may be more suitable for studying drugs that target MLVs. Second, MLV dysfunction appears to be potentially reversible within a specific time frame in pharmacological and surgical MLV models. For example, PDT-based MLV dysfunction can be repaired after 2 weeks, and MLVs must be ablated again to maintain malfunction [3]. This is possibly due to the compensatory mechanisms following g MLV blockage, including CSF outflow pathways, alternative lymphangiogenesis [2, 13], and an increase in MLV diameter without new lymphangiogenesis [88]. Third, the extent to which these models induce MLV dysfunction may differ. Mechanical ligation appears to completely obstruct MLV drainage in surgical models, whereas MLVs are only partially blocked in pharmacological and transgenic models. Specifically, no tracer was detected in the dCLNs of surgical models [3, 8, 10], whereas pharmacological and transgenic models caused tracer leakage in the dCLNs [3]. The similarities and differences between these models require further investigation.

### 3.2 *In Vitro* Models

Human lymphatic endothelial cells (HLECs) have been employed to mimic the physiology of MLVs [48]. The use of HLECs alone may lack features related to MLV functions, but utilizing HLECs in combination with other technical methods can help improve this *in vitro* model. First, the coculture of HLECs with other important components, such as peritoneal macrophages [14] and neuroblastoma cells [89], may improve the reliability of *in vitro* methods for studying MLV functions. Second, combining HLECs with PDT may be a viable option to better mimic MLV dysfunction *in vitro* [90]. PDT-based HLEC models have been successfully established in cancer research [91-93] and eye diseases [94-96]. This exploration has laid the groundwork for the establishment of the PDT-based *in vitro* model for MLVs. Third, the disturbance of brain water homeostasis represents a missing link in *in vitro* MLV and CSF drainage research. More research combining different *in vitro* techniques, such as the calcein fluorescence quenching method, an impedance-based microfluidic cell volume sensor, and a microfluidic volume sensor, may help solve the experimental challenges faced when measuring fluid disturbance *in vitro* [97-99].

## 4 HOW DO MLV_S_ REGULATE NEUROINFLAMMATION IN ND_S_?

### 4.1 Dysfunctional MLVs: Adverse Factors in the Pathogenesis of AD and PD

#### 4.1.1 Neurotoxic Protein Accumulation

The relationship between neurotoxic proteins and neuroinflammation has been well-established in AD and PD [100-102]. Typically, neurotoxic proteins lose their physiological roles and acquire neurotoxic properties, bind to pattern recognition receptors on microglia and astroglia, and initiate an innate immune response characterized by the release of inflammatory mediators [103, 104]. Considering the critical roles of MLVs in macromolecular drainage, MLV dysfunction might aggravate AD and PD and induce a pathogenic positive feedback loop between neurotoxic protein accumulation and neuroinflammation. Several recent studies have observed MLV dysfunction in mouse models of AD and PD. Compared with the control groups, Aβ [3, 8], tau [9], and α-synuclein [10, 54] were relatively high in the brains of mice with blocked MLV drainage.

**Fig. (2):**
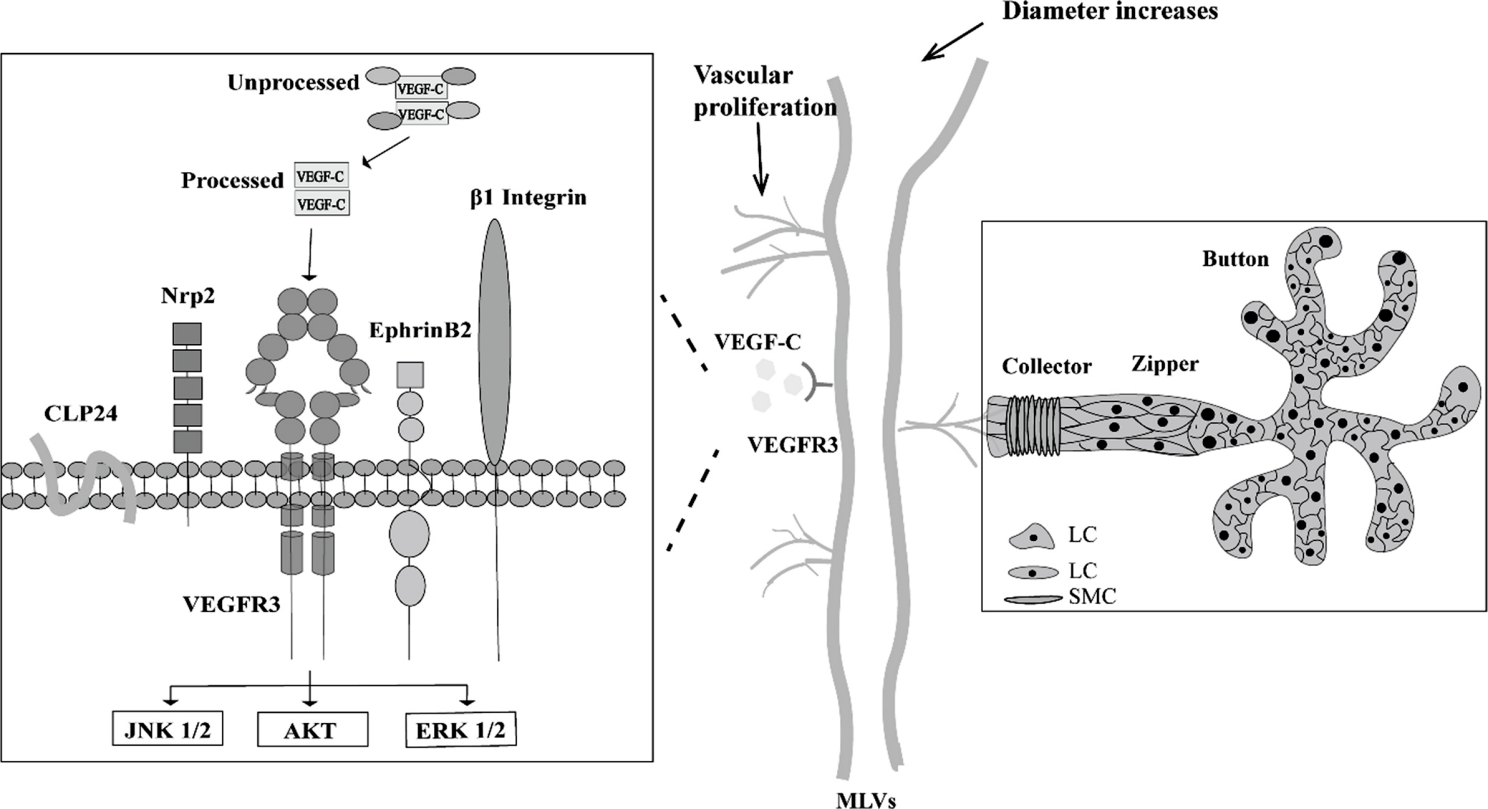
Role of the VEGF-C/VEGFR3 pathway in the regulation of MLVs. VEGF-C/VEGFR3 signaling, one of the primary pathways of MLV growth, has significant effects on MLV proliferation and diameter increase. VEGF-C forms homodimers or heterodimers with its structurally related receptor, VEGFR3. Membrane proteins, such as Nrp2, CLP24, b1 integrin, and EphrinB2, interact with VEGFR3 and VEGF-C and further activate various phosphorylation sites, leading to downstream signaling *via* the JNK1/2, ERK1/2, and AKT pathways. The illustration also depicts the different structures of MLVs. **Abbreviations:** AKT: protein kinase B; CLP24: Claudin-like protein 24; ERK 1/2: extracellular signal-regulated kinases 1/2; JNK 1/2: c-Jun N-terminal kinases 1/2; LC: lymphatic cells; MLVs: meningeal lymphatic vessels; Nrp2: neuropilin 2; SMC: smooth muscle cells; VEGF-C: vascular endothelial growth factor-C; VEGFR3: vascular endothelial growth factor receptor 3.

The VEGF-C/VEGFR3 pathway, a key factor for lymphatic endothelial cell growth, migration, and survival [105-108], has been identified as the most prevalent mechanism in MLV-related pathologies (Fig. **2**). Intracranial injection of VEGF-C induces dural lymphangiogenesis [48] and an increase in MLV diameter [1, 3] to accelerate the clearance of neurotoxic proteins. Notably, VEGF-C could only prevent Aβ deposition and cognitive impairment in aged animals [3]. This may be due to the cooperative ability of MLVs and the blood-brain barrier (BBB) to clear neurotoxic proteins. As the BBB’s clearance capacity deteriorates with age, meningeal lymphatic drainage of neurotoxic protein becomes inadequate. Future research should focus on elucidating how MLVs interact with BBB to clear neurotoxic proteins [7].

#### 4.1.2 Microglia Activation

Considering the fundamental role of microglia-derived inflammatory episodes in ADs, research on the involvement of dysfunctional MLVs in microglia activation is warranted. Evidence supports the idea that microglia activation signatures are shaped by dysfunctional MLVs. Transcriptome analysis of homeostatic and disease-associated microglia gene signatures from 5xFAD mice with ablated MLVs showed a strong association between impaired MLVs and microglial activation, with a high expression of genes involved in the transition from homeostatic to activated microglia and the acquisition of the neurodegenerative microglial signature [26]. Furthermore, microglia show similar activation signatures in the brains of patients with AD patients and AD transgenic mice with ablated MLVs, providing evidence that MLVs may play a role in modulating the neuroinflammatory response in AD [26]. Thus, it might be possible to devise strategies to therapeutically target microglia by modulating MLV function at the brain borders [26].

However, previous studies on the mechanisms underlying the link between microglia and MLVs have been carried out in the presence of neurotoxic proteins. Therefore, the extent to which MLV dysfunction directly impacts microglia activation, if at all, is unknown. To establish a direct MLV-mediated microglial reaction, a proper experimental model of the microglial inflammatory response must first be chosen. Lipopolysaccharide (LPS)-treated mice, one of the most common animal models of neuroinflammation and neurodegeneration, have shown extensive and prolonged microglial activation in different regions of the brain parenchyma [109, 110]. Typically, LPS binds to its specific ligand, toll-like receptor 4, which is expressed on the surface of microglia, triggering the downstream myeloid differentiation primary response protein 88-dependent and TIR-containing adaptor, inducing interferon β-dependent inflammatory cascades [111, 112]. LPS injection (1 mg/kg, intraperitoneally) in mice impaired perivascular CSF distribution without affecting the glymphatic system [113], one of the main components of the brain lymphatic drainage system [114]. LPS injection (1 mg/kg, intraperitoneally) in mice limits the interference of other factors that influence CSF drainage, making LPS-treated mice an ideal model for studying MLV-mediated microglia. However, LPS is potentially instrumental in the exacerbation of Aβ and tau pathology [115-118]. For example, after 6 days of receiving 1 mg/kg LPS intraperitoneally, a significant accumulation of Aβ precursor protein was found in the corpus callosum of adult Sprague Dawley rats [116]. Neurotoxic protein levels must be evaluated when using LPS to establish the direct MLV-mediated microglial reaction.

#### 4.1.3 Aquaporin 4 Expression

The CNS lymphatic transport system is composed primarily of the glymphatic system and the MLVs, with MLVs located downstream of the glymphatic system [63]. In the glymphatic system, the CSF flows into the brain along arterial perivascular spaces and subsequently into the brain interstitium. The CSF then flows toward the venous perivascular and perineuronal spaces, ultimately clearing solutes from the neuropil into the MLVs [62, 119]. Aquaporin 4 (AQP4), an aquaporin densely expressed in the astrocytic endfeet, is a key player in the glymphatic system. It facilitates the transport of CSF in the glymphatic system [62, 114, 119]. MLV malfunction was reported to be accompanied by impairment of AQP4 expression and polarization [10, 72]. For instance, dysfunctional MLVs may contribute to neuroinflammation by increasing AQP4 expression, thereby affecting Aβ accumulation in the brain [72]. However, studies have also reported that APQ4 is not associated with dysfunctional MLVs [3, 120]. A likely reason for these contradictory results is that the studies used different experimental disease models, which have unique intracerebral pathophysiology with or without neurotoxic protein accumulation. In addition, AQP4 plays an intrinsic proinflammatory role in the CNS [121, 122]. Thus, although AQP4 accelerates the outflow of neurotoxic proteins, its upregulated expression may lead to an exacerbated inflammatory reaction. More studies are needed to clarify the interaction between AQP4 and MLVs in NDs.

### 4.2 Dysfunctional MLVs: Protective Factors in MS and Stroke

Intriguingly, evidence indicates that dysfunctional MLVs exert an anti-neuroinflammatory effect in MS and stroke. In an experimental autoimmune encephalomyelitis mouse model of MS, both dCLN resection and MLV ablation lowered cluster of differentiation 4 (CD4) T cell infiltration of the spinal cord parenchyma, whereas they increased the number of CD4 T cells in the meninges, leading to milder neuropathology [11]. Similarly, dCLN resection reduced the activation of neutrophils and macrophages in both the blood and the brain, ameliorating brain injury in a middle cerebral artery occlusion rat model of stroke [14]. The downregulation of the CCR7/CCL21 and VEGF-C/VEGFR3 signaling pathways has been proposed as a core mechanism of inflammatory responses mediated by MLVs and dCLNs, playing a neuroprotective role in MS and stroke [11, 14, 31, 58, 72].

**Fig. (3):**
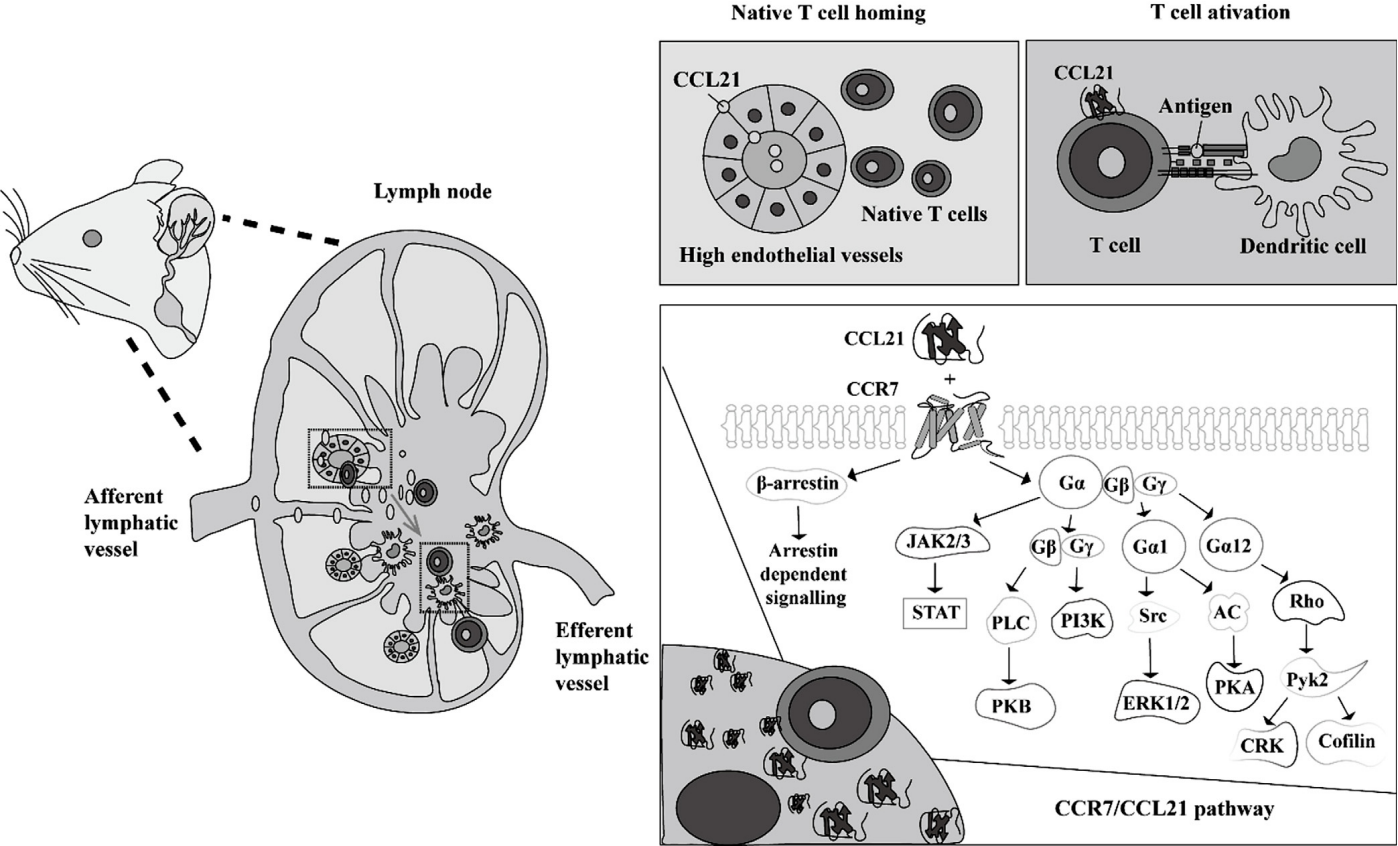
Role of the CCR7/CCL21 pathway in the regulation of meningeal immune cells to the dCLNs. MLVs are essential for immune cell drainage to the dCLNs, which is dependent on the CCR7/CCL21 pathway. In a typical scenario, antigen-carrying mature dendritic cells upregulate CCR7, a seven-fold transmembrane G protein-coupled receptor, and are home to dCLNs, which can activate T cells. CCL21, which is present in high endothelial venules, is thought to be the primary lymph node-homing chemokine, directing the lymph node localization of activated CCR7+ dendritic cells *via* afferent lymphatics. Subsequently, scanning and dendritic cell-T cell priming processes occur in the dCLNs. In the CCR7/CCL21 axis, CCL21 binds to CCR7, leading to conformational changes in the receptor and the subsequent detachment and separation of Gα and Gβ/Gγ subunits of the G-protein complex. A host of intracellular proteins are activated, resulting in the propagation of signaling cascades. **Abbreviations:** AC: adenyl cyclase; CCL21: chemokine (C-C motif) ligand 21; CCR7: CC-chemokine receptor 7; CRK: CT10 regulator of kinase; dCLNs: deep cervical lymph nodes; ERK 1/2: extracellular signal-regulated kinase 1/2; JAK2/3: janus kinase 2/3; PI3K: phosphoinositide 3-kinase; PKA: phosphokinase A; PKB: protein kinase B (Akt); PLC: phospholipase C; Pyk2: proline-rich tyrosine kinase 2; Rho: ras homolog; Src: proto-oncogene tyrosine-protein kinase; STAT: signal transducer and activator of transcription proteins.

A prerequisite for revealing the functions or roles of MLVs in MS and stroke is understanding the unique migratory behavior of immune cells in the meningeal spaces of the CNS [123, 124] (Fig. **3**). Recent research suggests that dCLNs represent important sites in which T cells develop a fully encephalitogenic profile [11]. When drainage is obstructed by the removal of dCLNs, the lack of lymphatic drainage reduces interactions between T cells and antigen-presenting cells [11]. Therefore, the activation of different T cell subsets is inhibited [76]. Because MS and stroke are characterized by reactive immune cells invading the brain [19, 125], the brain pathology of MS and stroke may be ameliorated when drainage from MLVs to dCLNs is blocked.

## 5 THE POTENTIAL ROLE OF NEUROINFLAMMATION IN STRUCTURAL AND FUNCTIONAL ASPECTS OF MLVs

### 5.1 Lymphangiogenesis

Lymphangiogenesis is a common pathophysiological phenomenon associated with inflammation [126-128]. Neuroinflammation is linked to meningeal lymphangiogenesis in stroke [12-14] and MS [129]. The inflammatory lymphangiogenesis of MLVs may act as functional compensation for the increased biological needs of neuroinflammation and could be beneficial for tissue homeostasis and repair [35, 130]. For instance, cerebrovascular injuries induce MLV ingrowth into the injured brain parenchyma after a stroke [12, 13]. These ingrown MLVs become lumenized to drain interstitial fluid from the injured parenchyma and resolve cerebral edema. In addition, they serve as a migratory scaffold to guide and support the growth of nascent blood vessels [13]. Furthermore, studies have suggested that VEGF-C, VEGFR2, VEGFR3, tyrosine kinase with immunoglobulin-like and epidermal growth factor-like domains 2, and delta-like canonical Notch ligand 4 play modulatory and facilitating roles in meningeal vascular regeneration, and these proteins have been proposed to be involved in the repair mechanisms for the restoration of MLV dysfunction [2, 3, 48, 131, 132]. However, in another report, the authors did not observe morphological changes in some specific areas of the MLVs during robust inflammation induced in an animal model of MS, such as spinal cord MLVs [11]. The limited lymphangiogenesis ability of MLVs may be attributed to dysregulated molecules involved in lymphatic development and proliferation, such as the Semaphorin 3A gene [133], as suggested by RNA-sequencing data from these experiments [11]. Elucidating the characteristics and molecular mechanism of neuroinflammation-induced lymphangiogenesis in different NDs will contribute to a comprehensive understanding of the structural and functional aspects of MLVs in brain injury recovery and the posterior development of NDs.

### 5.2 MLV Anomalies

MLV anomalies are a type of cerebrovascular anomaly, a term used to describe a variety of vascular lesions [88]. Cerebrovascular anomalies are typically aggravated by neuroinflammation induced by neurotoxic protein accumulation [134], systemic inflammation [135], and intracranial pressure (ICP) [136]. Considering the shared features of cerebrovascular and MLV anomalies [137], it is worth exploring the potential roles of neuroinflammation in MLV anomalies.

#### 5.2.1 Contribution of Neurotoxic Protein Accumulation

Neurotoxic protein accumulation has been viewed as the driver of severe neuroinflammation, which triggers cerebrovascular anomalies [138-140]. After exposure to exogenous neurotoxic proteins, endothelial cells and smooth muscle cells were surrounded by microglia, increasing pro-inflammatory cytokine expression and resulting in altered vasculature integrity, leakage, and infiltration [141]. Severe neuroinflammation caused by neurotoxic protein accumulation in the MLVs may be one of the most critical risk factors for MLV anomalies. One study found that the number of meningeal macrophages significantly increased one week after MLV ablation in 5xFAD mice compared with the control group [3], indicating severe inflammation following increased Aβ deposition in dysfunctional MLVs.

The genetic risk factors related to neurotoxic protein accumulation in NDs could reveal a missing link in our understanding of neuroinflammation in MLV anomalies. Apolipoprotein E4 (APOE4) increases the risk of AD by driving earlier and more abundant amyloid pathology in the brains of APOE*ε4 carriers [142]. The interaction between APOE4 and MLVs appears to play a role in AD [137]: APOE4 is likely linked to reduced expression of lymphatic vessel genetic markers and peripheral lymphedema-associated genes, and therefore, APOE4’s action could result in the early shrinkage and attenuated function of MLVs, diminishing CSF flow and decreasing Aβ clearance. As a result, APOE4 may exert some of its Aβ-related effects by inducing MLV anomalies. Future studies exploring the role of genetic risk factors related to neurotoxic protein accumulation in MLV anomalies may uncover promising avenues for the development of ND treatment.

#### 5.2.2 Stimuli from Systemic Inflammation

Systemic inflammation is a crucial factor in the treatment of chronic NDs. It is typically caused by a brain, oral, or gut infection, which can kill neurons and glia *via* nuclear factor kappa B-primed neuroinflammatory cascades [143, 144]. LPS frequently acts as a common proinflammatory stimulus in the CSF, aggravating cerebrovascular anomalies and inducing a potentially disruptive endothelial change in neuroinflammation [144, 145]. Intriguingly, researchers found that LPS exited the meningeal cells and co-localized with a series of inflammatory biomarkers, including cluster differentiation antigen 14, toll-like receptor 4, and the nuclear factor-kappa B [146]. Thus, LPS may cause vascular damage in MLVs and lead to their dysfunction. LPS dosage is a key factor to consider when LPS is used to induce MLV anomalies in experimental models. LPS doses have different effects on neuroinflammation [109]. A high dose of LPS (1 or 3 mg/kg) can cause cerebral microbleeds and may even disrupt the BBB *in vivo* [147, 148]. However, a low dose of LPS (0.2-0.9 mg/kg) was used to induce neuroprotective effects in immune training [149]. Thus, different LPS doses can have substantially different effects, and the appropriate dosage depends on the aim of the MLV model.

#### 5.2.3 Disturbance of ICP

ICP elevation has emerged as a major proinflammatory factor that contributes to impaired brain lymphatic drainage through astrocytic and microglia activation [150], and the subsequent inflammatory response may further impair brain lymphatic drainage [151]. Research suggests that elevated ICP-induced inflammation contributes to MLV anomalies. ICP elevation typically accompanies traumatic brain injury (TBI) [152], which has been associated with an increased risk of NDs, including AD, PD, and amyotrophic lateral sclerosis [153-155]. Studies in TBI mouse models have revealed that mild brain trauma can lead to severe deficits in meningeal lymphatic drainage that can last for at least one month [50, 51]. Mice with jugular vein ligation had impaired MLV function compared with mice that underwent sham surgery, which was associated with a transient elevation of ICP; in addition, MLV rejuvenation could improve TBI-driven neuroinflammation in mice [50]. These findings indicate that the transient increase in ICP could be involved in the mechanism of impaired meningeal lymphatic drainage.

In addition, MLV anomalies are likely caused by the combined action of acute ICP spikes, erythrocytes, immune cells, and debris. These confounding proinflammatory factors could impair MLVs directly in the context of subarachnoid hemorrhage [156]. Future research is needed to elucidate the precise effects of ICP disturbance and its complex mechanistic factors on MLV anomalies as well as the proinflammatory mechanisms that underpin these impacts.

## CONCLUSION AND FUTURE PERSPECTIVES

**Fig. (4):**
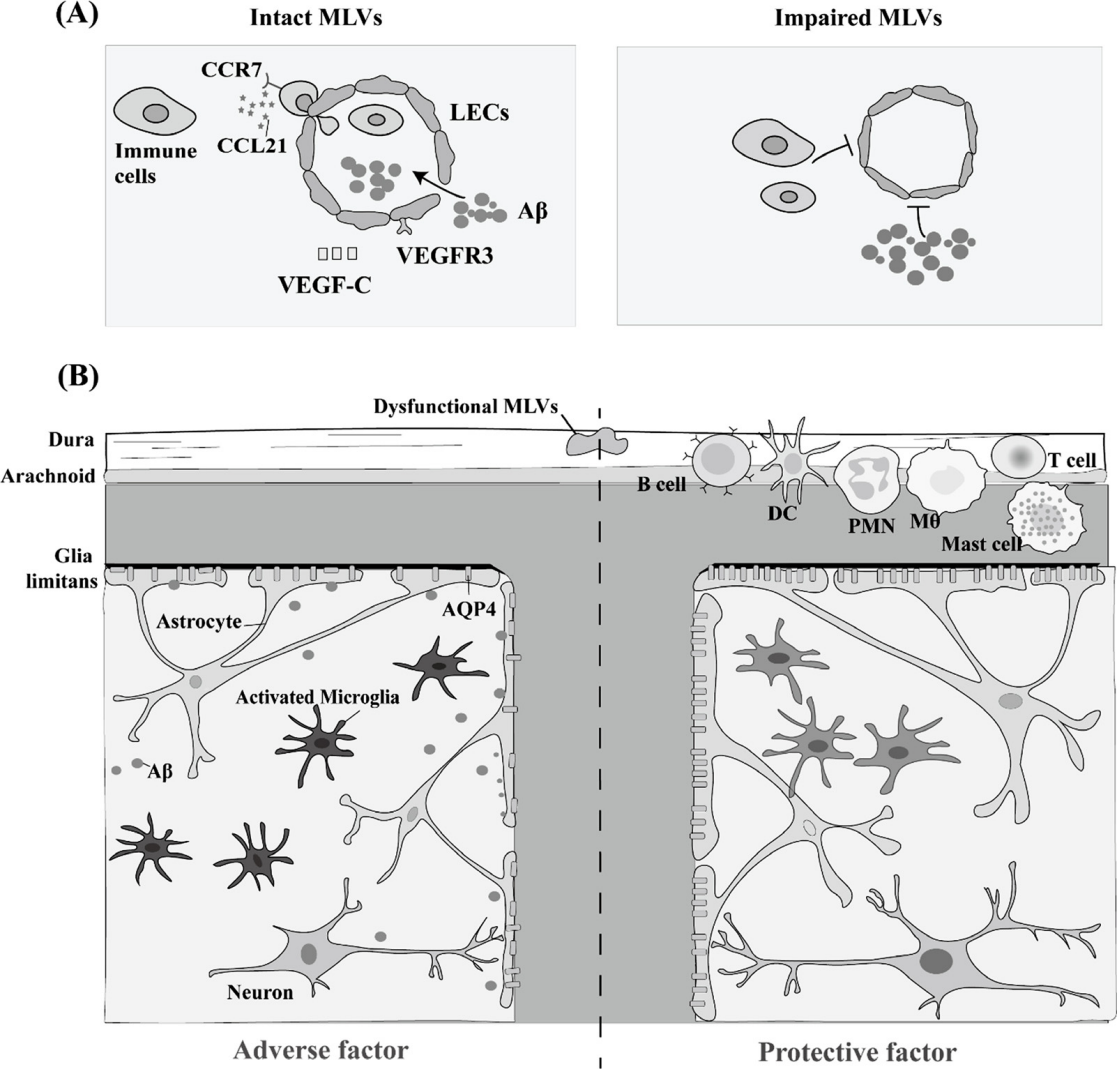
Dural effects of MLVs regulating neuroinflammation in NDs. (**A**) MLVs play a critical role in macromolecular drainage through the VEGF-C/VEGFR3 pathway. MLVs are also important for immune cell drainage *via* the CCR7/CCL21 pathway. (**B**) MLV dysfunction aggravates NDs that involve severe neurotoxic protein deposits, such as AD and PD. Besides, when MLV malfunction occurs, the ability of immune cells to develop an encephalitogenic profile in dCLNs is diminished. The reduction in MLV-mediated immune cell drainage ameliorates NDs that involve serious lymphocyte expansion in the meninges, such as MS. **Abbreviations:** Aβ: β-amyloid; AD: Alzheimer’s disease; AQP4: aquaporin 4; CCL21: chemokine (C-C motif) ligand 21; CCR7: CC-chemokine receptor 7; DC: dendritic cell; dCLNs: deep cervical lymph nodes; LEC: lymphatic endothelial cells; Mθ: macrophagocyte; MLVs: meningeal lymphatic vessels; MS: multiple sclerosis; NDs: neurodegenerative diseases; PD: Parkinson's disease; PMN: polymorphonuclear; VEGF-C: vascular endothelial growth factor C; VEGFR3: vascular endothelial growth factor receptor 3.

This review provides evidence supporting the role of the interaction between MLVs and neuroinflammation in NDs and proposes multiple research directions to advance our understanding of this fundamental process. Under pathological conditions, dysfunctional MLVs reduce the efficiency of toxic protein removal, aggravating neuroinflammation and accelerating ND progression. Conversely, MLV dysfunction can also provide anti-neuroinflammatory effects by preventing immune cells from developing an encephalitogenic profile (Fig. **4**). Furthermore, MLVs are influenced by neuroinflammation-induced lymphangiogenesis and cerebrovascular anomalies, which are attributed to neurotoxic protein accumulation, immune cell dysfunction, systemic inflammation, and ICP elevation. This framework offers promising opportunities for enhancing our understanding of the MLV-inflammation interplay in NDs. In addition, pathomechanistic responses that accompany the neuroinflammatory reaction in NDs should be considered in MLV research. Oxidative stress is closely related to the development of neuroinflammation in NDs [157], and reactive oxygen species, the extremely reactive intermediates of neuroinflammation, can cause brain injury [158, 159]. Evidence shows that reactive oxygen species may have a significant impact on lymphangiogenesis and MLV function [45, 160]. Natural products have both anti-inflammatory and antioxidative effects [161], and they may be beneficial in delaying the onset or progression of NDs by preventing MLV dysfunction. A deeper investigation into therapeutic interventions that modulate the interaction between MLVs and inflammation to improve cognition and reduce the risk of NDs is warranted.

## Figures and Tables

**Table 1:** MLV structure and function discovery.

**Year**	**Sample Source(s) and/or ** **Disease Model(s)**	**MLV Model(s)**	**MLV Detection Method(s)**	**MLV Structure and/or Function**	**References**
1787	Human	/	/	The first mention of LVs in the human brain meninges.	[37]
1953	Human	/	/	LVs exist in the human dura.	[38]
1960s	Dogs	/	/	A lymphatic connection exists between the CNS and the periphery.	[39]
1987	Rats	/	Electron microscope	Dural vessels appear to be LVs based on ultrastructural features.	[40]
1996	Human	/	Electron microscope	The round to oval stomata located between the mesothelial cells of the meninges appears to be LVs.	[41]
2000-2012	Autologous blood i.c.m induced SAH mouse model and MCAO rat model	dCLN resection	Indirect observational measures	Blocking cerebral lymphatic drainage aggravates cerebral injury in SAH and stroke.	[42-45]
From 2015 to the present	Human dura, C57BL/6 mice, Prox1^tdT^ mice, K14-VEGFR3-Ig mice, Prox1-GFP mice, and VEGFR3^+/LacZ^ mice	CLN resection/ligation and transgenic mice with LV deficiency	Confocal microscopy, fluorescent stereomicroscope, multiphoton microscopy, and flow cytometry	Rediscovery of MLVs serving the CNS.	[1, 5]
	Nonhuman primates	/	MRI	MLVs exist in common marmoset monkeys.	[46]
	Human dura	/	MRI	Further confirmation of the existence of human MLVs.	[46, 47]
	Human dura, J20 mice, 5xFAD mice, Prox1^LacZ^ mice, and APP/PS1 mice	PDT, dCLN ligation, and transgenic mice with LV deficiency	MRI, flow cytometry, confocal microscopy, and RNA sequencing	MLVs regulate Aβ clearance in the CNS.	[3, 8, 48]
	Experimental autoimmune encephalomyelitis mouse model and Prox1^Het^ mice	PDT, dCLN resection/ ligation, and transgenic mice with LV deficiency	Multiphoton microscopy, RNA sequencing, and flow cytometry	MLVs govern inflammatory processes and immune surveillance of the CNS.	[11]
	A53T mice	dCLN ligation	Fluorescence microscope and indirect observational measures	MLV dysfunction exacerbates α-synuclein accumulation in PD.	[10]
	K14-VEGFR3-Ig mice	Transgenic mice with LV deficiency	Fluorescence molecular tomography	MLVs regulate tau clearance in the CNS.	[9]
	Mouse model of stroke induced by photothrombosis, zebrafish model of cerebrovascular injury using genetic ablation and photochemical thrombosis, VEGFR3^wt/wt^ mice, and VEGFR3^wt/mut^ mice	Transgenic mice with LV deficiency	3D imaging and live imaging	Cerebrovascular injury after stroke induces the growth of MLVs into the injured brain parenchyma.	[12, 13]
	MCAO rat and mouse models	VEGFR3 inhibition and sCLN resection	Immunofluorescence	MLVs ameliorate systemic inflammation and brain injury after stroke.	[14]
	Glioblastoma multiforme mouse model	dCLN ligation	Confocal microscopy and flow cytometry	VEGF-C-driven MLV drainage enables brain tumor immunosurveillance.	[32]
From 2015 to the present	Patients with cerebrospinal fluid disorders	/	MRI	Parasagittal dura may serve as a bridging link between the human brain and dural MLVs.	[49]
	Autologous blood i.c.m induced SAH mouse model	PDT and VEGFR3 inhibition	Indocyanine green near-infrared imaging and confocal microscope	MLVs drain erythrocytes to the CLNs after SAH.	[25]
	‘Hit & Run’ model of closed-skull traumatic brain injury	PDT	Immunofluorescence	Increased intracranial pressure impairs MLVs.	[50, 51]
	Zebrafish	/	High-resolution optical imaging in living animal	Zebrafish possess a meningeal lymphatic network.	[52]
	Human brain and ganglion	/	Multiplex immunostainings	Lymphatic endothelial cells have been visualized in the human brain, meninges, cavernous sinus, and cranial nerves.	[53]
	Patients with iPD	/	MRI	Lymphatic flow through MLVs is significantly reduced in patients with iPD compared to patients with AP.	[54]
	5xFAD mice	PDT	Bulk RNA sequencing	MLVs affect microglia responses and anti-Aβ immunotherapy.	[26]
	Experimental autoimmune encephalomyelitis mouse model	/	Single-cell RNA sequencing, MRI, and flow cytometry	The MLVs near the cribriform plate regulate immunity.	[55]
	Mice injected with senescent astrocytes	dCLN ligation	Immunofluorescence	MLVs drain senescent astrocytes.	[56]
	Virus-infected mice	PDT and dCLN ligation	Three-dimensional fluorescence imaging, flow cytometry, *in vivo* animal imaging, and two-photon imaging	MLVs drain neurotropic virus to the CLNs.	[57]

**Abbreviations:** Aβ: β-amyloid; AD: Alzheimer’s disease; AP: atypical Parkinsonian; CNS: central nervous system; CLNs: cervical lymph nodes; dCLNs: deep cervical lymph nodes; iPD: idiopathic Parkinson's disease; LVs: lymphatic vessels; MLVs: meningeal lymphatic vessels; MCAO: middle cerebral artery occlusion; MRI: magnetic resonance imaging; MS: multiple sclerosis; PD: Parkinson's disease; PDT: photodynamic therapy; SAH: subarachnoid hemorrhage; sCLNs: superficial cervical lymph nodes; VEGF-C: vascular endothelial growth factor-C; VEGFR3: vascular endothelial growth factor receptor 3; /: not available.

**Table 2:** Mechanisms of dysfunctional MLVs in different animal models.

**Model**	**Examples**	**Evidence of MLV Dysfunction**	**Possible Mechanisms**
Surgical model	● C57BL/6 mice with dCLN ligation [3] ● APP/PS1 mice with dCLN ligation [8] ● A53T mice with dCLN ligation [10] ● Prox1^Het^ mice with dCLN ligation [11] ● MCAO rat model with dCLN resection [42-44] ● MCAO mouse model with sCLN resection [14]	Effect demonstrated with: ● CSF and ISF [3] ● Aβ [8] and α-synuclein [10] ● Immune cells [11, 14]	● MLVs are anatomically connected to sCLNs and dCLNs. ● dCLNs are important sites for T cells to develop a fully encephalitogenic profile.
Pharmacological model	● C57BL/6 mice with PDT [3] ● J20 transgenic mice with PDT [3] ● MS mouse model with PDT [11] ● SAH mouse model with PDT [25] ● VEGFR tyrosine kinase inhibitor-treated mice [2, 14, 25, 86] ● VEGF-C trap-treated mice [2]	Effect demonstrated with: ● CSF and ISF [3] ● Aβ [3] ● Immune cells [11] ● Erythrocytes [25]	● PDT produces highly reactive singlet oxygen to exert a cytotoxic effect. ● MLVs in adult mice is uniquely dependent on VEGF-C/VEGFR3 signaling.
Transgenic mouse model	● Prox1^+/−^ mice [3] ● Prox1^GFP^ mice [11] ● K14-VEGFR-3-Ig mice [9]	Effect demonstrated with: ● CSF and ISF [3] ● Tau [9] ● Immune cells [11]	● In such transgenic mouse model, abnormal expression of the main lymphatic endothelial cell transcription factor is related to defective MLVs.

**Abbreviations:** Aβ: β-amyloid; CSF: cerebrospinal fluid; dCLNs: deep cervical lymph nodes; ISF: interstitial fluid; MCAO: middle cerebral artery occlusion; MLVs: meningeal lymphatic vessels; MS: multiple sclerosis; PDT: photodynamic therapy; SAH: subarachnoid hemorrhage; sCLNs: superficial cervical lymph nodes; VEGF-C: vascular endothelial growth factor-C; VEGFR3: vascular endothelial growth factor receptor 3.

## LIST OF ABBREVIATIONS

AD: Alzheimer’s Disease
BBB: Blood-brain Barrier
CNS: Central Nervous System
CSF: Cerebrospinal Fluid
dCLNs: Deep Cervical Lymph Nodes
HLECs: Human Lymphatic Endothelial Cells
ISF: Interstitial Fluid
MLVs: Meningeal Lymphatic Vessels
MS: Multiple Sclerosis
PD: Parkinson’s Disease
PDT: Photodynamic Therapy
VEGFR: Vascular Endothelial Growth Factor Receptor
